# A Systematic Review of Personality Disorders in Patients with Gambling Disorder

**DOI:** 10.3390/clinpract16010015

**Published:** 2026-01-09

**Authors:** Ioana Ioniță, Mădălina Iuliana Mușat, Bogdan Cătălin, Constantin Alexandru Ciobanu, Adela Magdalena Ciobanu

**Affiliations:** 1 Faculty of Medicine, “Carol Davila” Doctoral School, University of Medicine and Pharmacy, 020021 Bucharest, Romania; ioana_ionita@ymail.com; 2 Experimental Research Center for Normal and Pathological Aging, University of Medicine and Pharmacy of Craiova, 2 Petru Rareș Street, 200349 Craiova, Romania; 3 Department of Scientific Research Methodology, University of Medicine and Pharmacy of Craiova, 2 Petru Rareș Street, 200349 Craiova, Romania; 4 Department of Physiology, University of Medicine and Pharmacy of Craiova, 2 Petru Rareș Street, 200349 Craiova, Romania; 5 Faculty of Medicine, “Carol Davila” University of Medicine and Pharmacy, 020022 Bucharest, Romania; alexandru.ciobanu2020@stud.umfcd.ro; 6 Department of Psychiatry, “Prof. Dr. Alexandru Obregia” Clinical Hospital of Psychiatry, 041914 Bucharest, Romania; adela.ciobanu@umfcd.ro; 7 Neuroscience Department, Discipline of Psychiatry, Faculty of Medicine, “Carol Davila” University of Medicine and Pharmacy, 020021 Bucharest, Romania

**Keywords:** gambling disorder, pathological gambling, personality disorder, impulsivity, comorbidity, psychopathology

## Abstract

Background/Objectives: Gambling disorder (GD) is characterized by a high prevalence of co-occurring psychiatric disorders, including personality disorders (PDs), which may negatively influence clinical presentation, treatment outcomes, and relapse rates. The aim of this systematic review was to synthesize recent evidence regarding the association between GD and formally diagnosed PD and/or diagnostically anchored PD symptomatology, and to describe the main personality dimension most frequently reported in affected individuals. Methods: A systematic search was conducted in the PubMed and Dialnet databases for articles published between 30 November 2015 and 30 November 2025, according to the Preferred Reporting Items for Systematic Reviews and Meta-Analyses (PRISMA 2020) guidelines. PubMed was selected as the primary database because it is the most comprehensive source for peer-reviewed biomedical and psychiatric research, while Dialnet was included to complement PubMed by ensuring coverage of peer-reviewed psychiatric and psychological research published in other Romance-language journals, which are often underrepresented in international databases. The methodological quality and risk of bias of the included studies were evaluated using the Joanna Briggs Institute (JBI) Critical Appraisal Checklist for cross-sectional studies and the Newcastle–Ottawa Scale (NOS) for observational studies. Data extraction and synthesis were performed manually by two independent reviewers. Eight studies, predominantly cross-sectional in nature, assessing exclusively formally diagnosed personality disorders in adult individuals (≥18 years) diagnosed with GD were included. Results: Eight studies met the inclusion criteria, including a total of 4607 patients with GD. Across studies, personality pathology was highly prevalent among individuals with GD, with antisocial and borderline personality disorders most consistently reported. Elevated levels of impulsivity, emotional dysregulation, and narcissistic traits were frequently observed and were additionally associated with greater gambling severity, earlier onset, and poorer clinical outcomes. Antisocial personality symptoms were strongly linked to high-risk gambling subtypes, while obsessive–compulsive personality traits showed a more heterogeneous relationship with gambling severity. Conclusions: These results underscore the importance of personality assessment in individuals with GD and highlight the need for longitudinal studies using standardized diagnostic frameworks to inform tailored prevention and treatment strategies.

## 1. Introduction

Gambling disorder (GD) has been recognized as a significant public health concern, with increasing prevalence worldwide and substantial personal, social, and economic consequences [1,2,3]. A growing body of research has highlighted the complex clinical profile associated with this pathological gambling (PG), including frequent psychiatric comorbidities [4], mood disorders [5,6,7,8], substance use disorders [9,10], and impulse-control disturbances [11,12], which complicate both diagnosis and treatment [13,14]. Among these comorbidities, personality disorders (PDs) appear to play a particularly important role in shaping the onset, course, and severity of gambling-related problems [15,16,17].

Personality pathology has been associated with heightened vulnerability to addictive behaviors [18,19], impaired emotional regulation [20], dysfunctional coping strategies [21], and reduced treatment adherence [22,23].

Traits such as impulsivity, emotional instability, sensation seeking, and interpersonal dysfunction have repeatedly been linked to problematic gambling behavior, suggesting that underlying personality dimensions may contribute to both the development and maintenance of GD [24,25].

Although numerous studies have examined personality traits in individuals with gambling disorder [26,27,28,29,30], the existing literature remains limited in its ability to provide a clear picture of formally diagnosed personality disorders in this population. Therefore, a systematic appraisal of recent research is required to clarify the relationship between gambling disorder and personality disorders, and to provide an updated overview of the most relevant findings.

In this context, a systematic synthesis of recent evidence is warranted to clarify the association between GD and formally diagnosed PD and/or diagnostically anchored PD symptomatology. The present systematic review aims to summarize and critically appraise studies published over the past decade that have examined personality pathology in adults with gambling disorder. Specifically, this review seeks to identify the most frequently reported formally diagnosed PD and/or diagnostically anchored PD symptomatology in this population, to explore their associations with gambling severity and clinical outcomes, and to highlight implications for assessment, and future research.

## 2. Materials and Methods

### 2.1. Protocol and Reporting Standards

A systematic review was conducted following the Preferred Reporting Items for Systematic Reviews and Meta-Analyses (PRISMA) guidelines [31]. A completed PRISMA checklist is provided in the Supplementary Materials (File S1).

The protocol was registered in Open Science Framework available at Registration DOI: 10.17605/OSF.IO/2B9NU.

### 2.2. Information Sources and Search Strategy

Relevant publications indexed in the electronic databases PubMed and Dialnet between 30 November 2015 and 30 November 2025 were examined. The search strategy employed the following combination of keywords: “gambling disorder” or “pathological gambling” AND “personality disorder”. PubMed was selected as the primary database because it provides the most comprehensive coverage of peer-reviewed biomedical and psychiatric literature, with standardized indexing and strong representation of studies using formal diagnostic frameworks. Dialnet was included as a complementary source to ensure the identification of peer-reviewed psychiatric and psychological research published in Romance-language journals, which are frequently underrepresented in major international databases. The inclusion of Dialnet therefore broadened the linguistic and geographical scope of the review while remaining consistent with its clinical and diagnostic focus.

### 2.3. Eligibility Criteria

**Figure 1:**
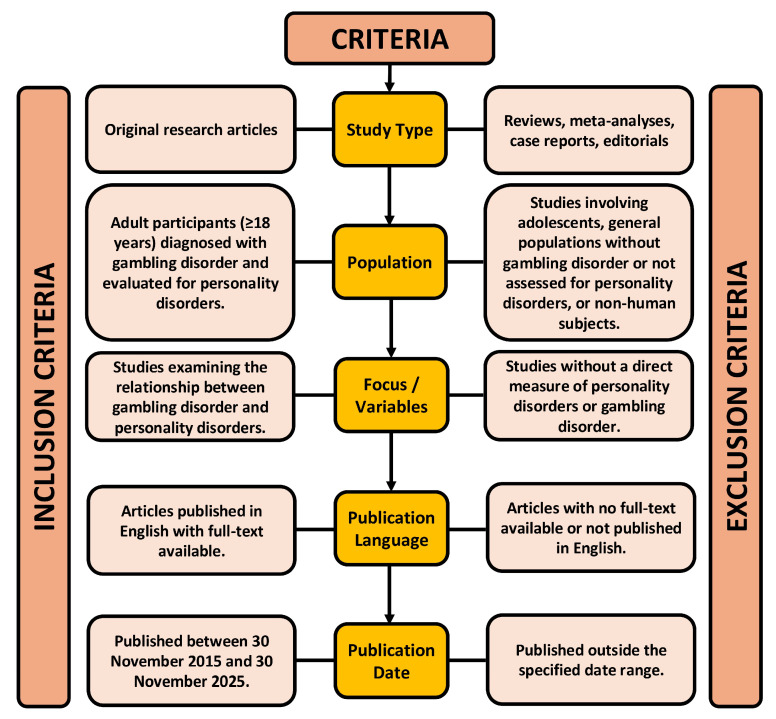
Inclusion and exclusion criteria.

The selection of studies was guided by clearly defined inclusion and exclusion criteria to ensure methodological rigor (Figure 1). These criteria were applied throughout the screening process in order to systematically identify research meeting the predefined objectives of the review, while excluding studies whose design, or population did not align with the established scope. As the objective of this review was to examine the association between gambling disorder and formally diagnosed PDs and/or diagnostically anchored PD symptomatology, rather than broader or dimensional personality constructs, studies focusing only on personality traits, impulsivity/disinhibition, emotion dysregulation, pathways model subtypes, or related transdiagnostic features were intentionally excluded unless a personality disorder was explicitly reported.

### 2.4. Study Selection and Data Extraction and Synthesis

Two reviewers independently screened the titles and abstracts of all retrieved records according to predefined inclusion and exclusion criteria. The study selection followed PRISMA 2020 guidelines. First, relevant records were identified through database searches and exported for review. Duplicate entries were then removed. Titles and abstracts were screened, and non-eligible studies were excluded. Full texts of the remaining articles were assessed to confirm eligibility, and finally, studies meeting all criteria were included. Discrepancies between reviewers at any stage of the selection process were resolved through discussion, with involvement of a third reviewer when required.

Data extraction and synthesis were performed independently by two reviewers and included the following variables: (1) author, year, country, study design; (2) sample size, sex distribution and age; (3) GD diagnostic criteria; (4) type of gambling; (5) personality assessment tool; (6) instrument description; (7) other measures applied; (8) main findings/key outcomes related to PD-GD relationship.

### 2.5. Risk of Bias and Quality Assessment

The methodological quality and risk of bias of the cross-sectional studies were evaluated using the Joanna Briggs Institute (JBI) Critical Appraisal Checklist for Analytical Cross-Sectional Studies [32], by two reviewers to minimize bias, with disagreements resolved by consensus. The checklist examines eight key methodological domains: (Q1) clearly defined inclusion criteria; (Q2) comprehensive description of study participants and setting; (Q3) valid and reliable assessment of exposure; (Q4) application of objective and standardized criteria for condition measurement; (Q5) identification of relevant confounding factors; (Q6) stated strategies to address confounding; (Q7) valid and reliable outcome measurement; and (Q8) use of appropriate statistical analyses.

The methodological quality of the observational studies was evaluated using the Newcastle–Ottawa Scale (NOS), which assesses three core domains: selection (up to 4 points), comparability (up to 2 points), and outcome (up to 3 points). Total scores of 7–9 were considered indicative of high quality, scores of 5–6 of moderate quality, and scores below 5 of low quality.

## 3. Results

### 3.1. Study Selection Process

**Figure 2:**
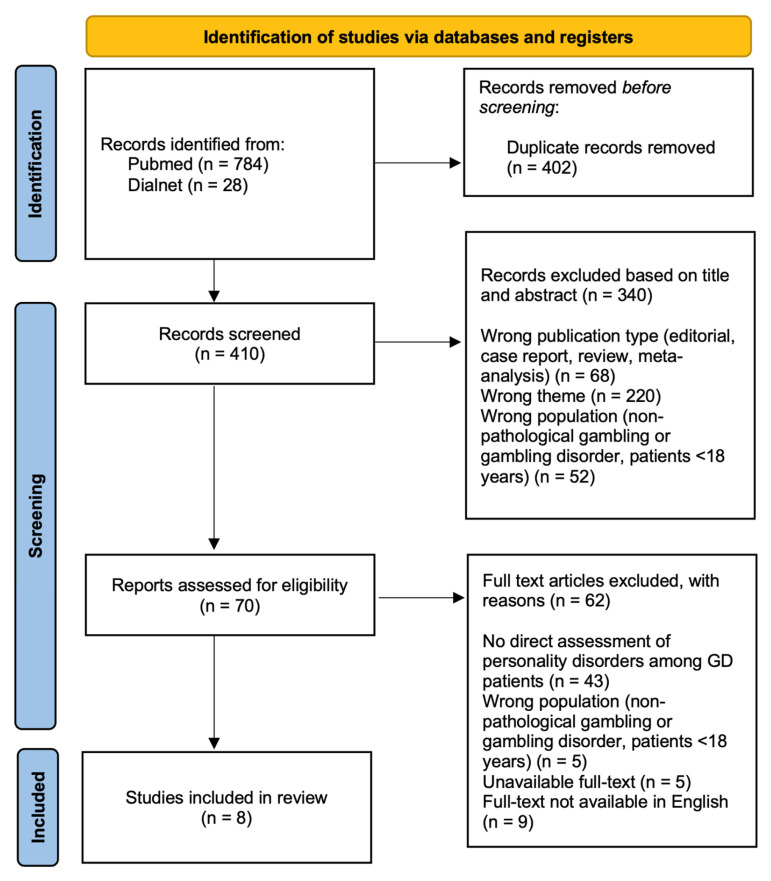
PRISMA flow diagram of the literature screening and selection process.

A total of 812 records were identified through the initial database search. Because identical articles were often indexed with slightly different metadata (e.g., variations in title formatting, language versions, or indexing fields) both within and across the two databases, the deduplication process yielded a substantial number of overlapping records, resulting in the removal of 402 duplicate entries. 410 records remained and were screened based on titles and abstracts. During the screening phase, 340 records were excluded. Following this step, 70 full-text articles were assessed for eligibility. Of the 70 full-text articles assessed for eligibility, 62 were excluded primarily because they did not examine formally diagnosed PD and/or diagnostically anchored PD symptomatology. Most of these studies focused on personality traits or dimensional personality characteristics that were not formally assessed using validated diagnostic instruments or established diagnostic criteria. As the present review was intentionally restricted to studies addressing diagnosed or diagnostically anchored PDs in individuals with GD, such studies were excluded despite their conceptual relevance. Ultimately, 8 studies met the inclusion criteria and were included in the final review, as illustrated in the PRISMA flow diagram (Figure 2).

### 3.2. Study Characteristics

**Figure 3:**
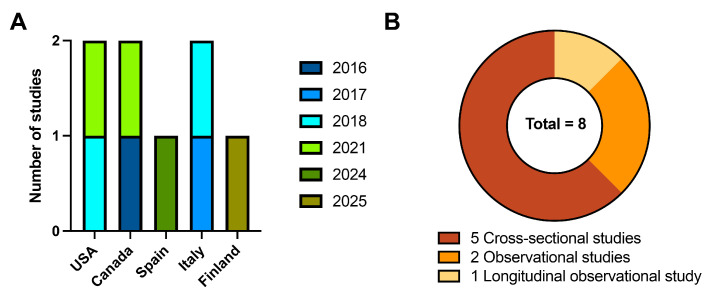
Distribution of the included studies by (**A**) country and year of publication and (**B**) study design.

A total of 8 studies were included in the review, published between 2016 and 2025, and conducted across North America (USA, Canada) and Europe (Italy, Spain, Finland) (Figure 3A). Most studies employed a cross-sectional design, while 3 studies used an observational design (Figure 3B).

Sample sizes varied considerably, ranging from 50 participants to 3605 individuals, comprising a total of 4607 patients with GD. The samples across studies were predominantly male, ranging from 50% to over 90%, which should be considered when interpreting the findings, as gender differences in gambling disorder and personality pathology have been previously reported.

Mean participant age across studies generally fell within mid-adulthood, ranging approximately from the mid-30s to late 40s.

Diagnostic criteria for gambling disorder included Diagnostic and Statistical Manual for Mental Disorders IV (DSM-IV), DSM-5, International Classification of Diseases, Tenth Revision (ICD-10), and validated screening instruments such as the South Oaks Gambling Screen (SOGS). Across studies, DSM-IV, DSM-5, or ICD-10 criteria were applied, reflecting both differences in publication period and the country-specific diagnostic systems in use at the time of data collection.

Gambling activities varied across studies and included slot machines, sports betting, card games, lotteries, and online gambling, with several studies distinguishing between strategic and non-strategic gambling types. The main characteristics of the studies included in the systematic review are summarized in Table 1.

### 3.3. Formally Diagnosed PD and/or Diagnostically Anchored PD Symptomatology Assessment in GD and Main Findings

Across the included studies, a consistent association emerged between GD and the presence of PDs or diagnostically anchored personality pathology. Studies using structured clinical interviews or DSM-/ICD-aligned diagnostic instruments reported high rates of comorbid PDs among individuals with GD. Medeiros et al. (2018) found that individuals with GD and comorbid Obsessive–Compulsive Personality Disorder (OCPD) showed lower gambling severity and a slower progression from recreational gambling to GD compared to those without OCPD [33]. Despite this, the GD + OCPD group still exhibited clinically significant gambling problems. This group also reported a greater number of gambling triggers, including money availability, stress, loneliness, and advertising exposure. Additionally, individuals with GD and OCPD demonstrated a higher prevalence of substance use disorders, suggesting a complex comorbidity profile (Table 2).

Salonen et al. (2025) reported that 7.9% of individuals with GD had a diagnosed adult PD, based on national healthcare registry data [36]. PDs showed the highest standardized incidence ratio (SIR = 616.6) among all psychiatric comorbidities associated with GD. The incidence was particularly elevated in women (SIR = 839.0), though it was also markedly high in men (SIR = 678.6). These findings indicate that PDs represent one of the strongest psychiatric correlates of GD at the population level. However, Quilty et al. (2021) demonstrated that individuals with problem gambling exhibited elevated symptoms across multiple PDs, regardless of the assessment method used [37]. The most prominent elevations were observed for Borderline, Paranoid, Schizotypal, Avoidant, and Dependent personality disorders. In addition, higher levels of Antisocial, Narcissistic, and Obsessive–Compulsive personality traits were identified. These findings support the presence of broad and multidimensional personality pathology among individuals with gambling problems (Table 2).

Regarding antisocial personality disorder (ASPD), Moon et al. (2016) found that antisocial personality disorder symptoms were most pronounced in the Antisocial–Impulsivist (AI) gambling subtype [39]. This subgroup was characterized by elevated impulsivity, increased risk-taking behavior, and higher levels of ADHD symptoms. The results suggest that antisocial personality traits play a central role in distinguishing a high-risk gambling phenotype marked by impulsivity and behavioral dysregulation.

On the other hand, Black et al. (2021) reported that ASPD symptoms were most prevalent in the Antisocial Drinker (AD) and A) gambling subtypes [40]. The AI subtype exhibited the highest levels of impulsivity, ADHD symptoms, and also increased gambling severity (Table 2).

Among studies that utilized validated diagnostic instruments for diagnostically anchored PD symptomatology assessment, Fierro et al. (2024) reported that individuals with GD scored significantly lower than the general population on several personality disorder dimensions, including paranoid, histrionic, narcissistic, passive-aggressive, and sadistic symptoms [34]. Within the GD group, higher antisocial and borderline personality symptoms predicted lower levels of physical activity, whereas higher obsessive–compulsive and self-destructive symptoms were associated with moderate to high levels of physical activity. These findings suggest that distinct personality profiles among GD patients are differentially related to health-related behaviors (Table 3).

Maniaci et al. (2017) found a high prevalence of personality disorders among individuals with GD, with 59.8% meeting criteria for at least one personality disorder and 25.6% presenting two or more personality disorders [35]. Compared to control participants, problem gamblers scored significantly higher on Narcissistic, Antisocial, Negativistic (Passive-Aggressive), and Self-Defeating personality disorders. Importantly, Antisocial and Negativistic personality disorders significantly predicted early treatment dropout, along with comorbid post-traumatic stress disorder and drug dependence, whereas anxiety symptoms acted as a protective factor against premature dropout. Rogier et al. (2018) demonstrated that individuals with GD exhibited significantly elevated levels of both grandiose and vulnerable pathological narcissism compared to non-gambling controls [38]. Grandiose narcissism was a significant predictor of gambling severity, with emotion dysregulation fully mediating this relationship. Vulnerable narcissism showed a strong association with emotion regulation difficulties. Additionally, strategic gamblers displayed higher levels of devaluing, a core feature of vulnerable narcissism, compared to non-strategic gamblers, highlighting the role of narcissistic pathology in gambling behavior (Table 3).

Prevalence estimates of PDs varied substantially depending on the assessment approach, with one study relying on multiaxial inventory in accordance with DSM-IV-TR in patients reported high prevalence rates, with up to 59.8% of individuals with GD meeting criteria for at least one personality disorder and approximately one-quarter presenting with multiple personality disorder diagnoses [35]. In contrast, registry-based data yielded lower absolute prevalence estimates (7.9%), but demonstrated markedly elevated incidence rates relative to the general population, particularly among women [36]. This pattern suggests systematic differences in prevalence estimates as a function of assessment method and sampling context.

### 3.4. Risk of Bias and Quality Assessment of the Studies Included

**Figure 4:**
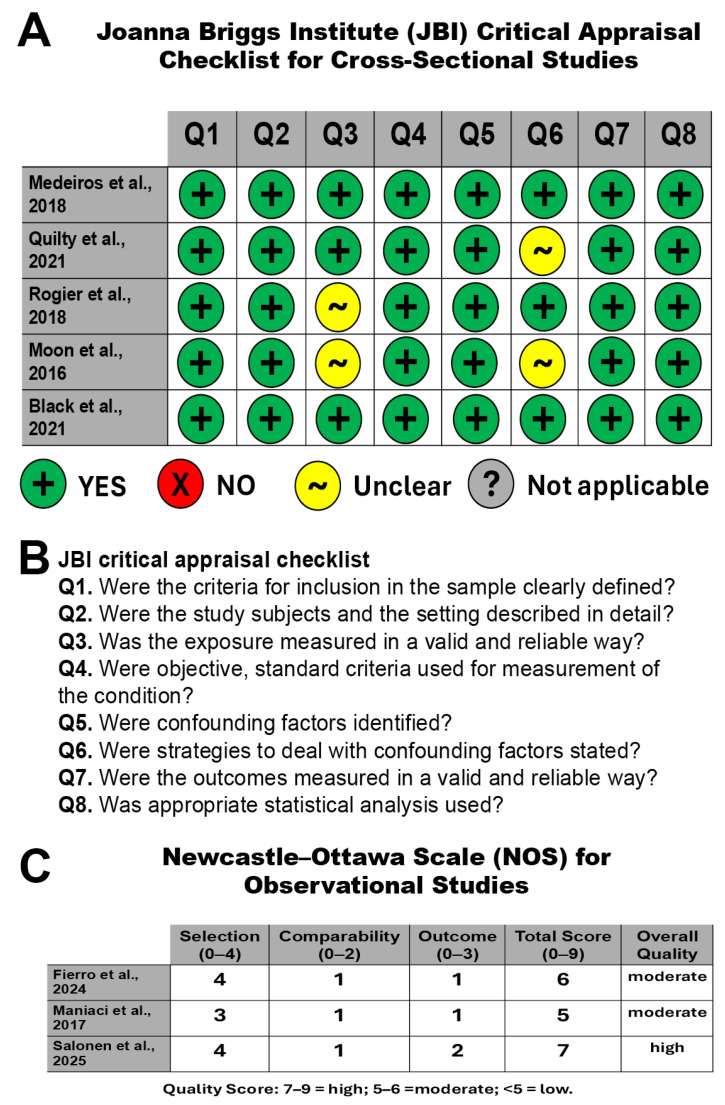
Risk of bias and methodological quality assessment of included studies. (**A**) Summary of the methodological quality assessment of the cross-sectional studies using the Joanna Briggs Institute (JBI) Critical Appraisal Checklist for Analytical Cross-Sectional Studies. Each row represents an individual study and each column corresponds to one checklist item (Q1–Q8). Symbols indicate appraisal outcomes: + = yes, x = no, ~ = unclear, and ? = not applicable [33,37,38,39,40]. (**B**) JBI critical appraisal checklist items. (**C**) Newcastle–Ottawa Scale (NOS) for observational studies. Overall methodological quality was classified as high (7–9 points), moderate (5–6 points), or low (<5 points) [34,35,36].

The methodological quality of the cross-sectional studies was assessed using the JBI Critical Appraisal Checklist for Analytical Cross-Sectional Studies (Figure 4A,B) and the NOS for the observational studies (Figure 4C).

The methodological assessment revealed that the included studies were of acceptable to high quality, with consistent strengths in study design, outcome measurement, and statistical analysis. The main methodological limitations were related to incomplete reporting of exposure validity and confounding control, which should be considered when interpreting the findings.

Overall, the included cross-sectional studies demonstrated good methodological quality, with most checklist items rated positively across studies. All studies clearly defined their inclusion criteria (Q1) and provided an adequate description of the study participants and settings (Q2). Furthermore, objective and standard criteria were consistently used for the measurement of the studied conditions (Q4), and all studies employed appropriate statistical analyses (Q8). Assessment of exposure measurement (Q3) was rated as unclear in two studies, reflecting limited reporting on the validity or reliability of the exposure instruments used. In addition, although confounding factors were generally identified (Q5), strategies to address confounding (Q6) were not consistently described, resulting in unclear ratings for this item in some studies. Importantly, outcome measures were largely assessed using valid and reliable methods across all included studies (Q7).

Using the NOS, total quality scores ranged from 5 to 7 points (out of a maximum of 9), indicating moderate to high methodological quality among the included observational studies. One study achieved a high-quality rating (7 points), reflecting strong performance in selection and outcome domains, supported by robust data sources and appropriate analytical methods. The remaining two studies were classified as moderate quality (5–6 points). In these studies, lower scores were mainly attributable to limited comparability and less robust outcome assessment, rather than major flaws in study design or execution. Overall, no study was classified as low quality, supporting the inclusion of all studies in the qualitative synthesis.

## 4. Discussion

### 4.1. Interpretation of the Results

The findings of this systematic review indicate a substantial overlap between GD and personality pathology, reinforcing the notion that GD is frequently embedded within a broader framework of maladaptive personality traits and psychiatric vulnerability. Personality pathology, particularly antisocial and borderline disorders, emerged as the most consistently reported pattern among individuals with GD. This finding is in strong concordance with prior review-based and systematic research, which has repeatedly highlighted the prominent role of maladaptive personality features in this population [15,41,42].

Antisocial personality disorder symptoms were strongly associated with higher gambling severity, impulsivity, risk-taking behavior, and comorbid ADHD symptoms [34,35,37,39,40]. These findings are congruent with theoretical models emphasizing impulsivity and behavioral disinhibition as core mechanisms underlying both antisocial traits and addictive behaviors [43,44,45]. Studies employing subtype or latent class analyses further demonstrated that antisocial–impulsivist gambling subtypes represent a clinically severe group characterized by early onset, poor emotional regulation, and adverse functional outcomes [40].

Borderline personality disorder symptoms were also frequently observed and appeared closely linked to emotional dysregulation, interpersonal instability, and heightened sensitivity to stress-related gambling triggers [34,37]. These traits may increase reliance on gambling as a maladaptive coping strategy, particularly in response to negative affective states. Similarly, narcissistic personality pathological variations, both grandiose and vulnerable, were significantly elevated among individuals with GD [38]. Notably, grandiose narcissism directly predicted gambling severity, with emotion dysregulation acting as a mediating mechanism, highlighting the central role of affective processes in the PD–GD relationship [38].

OCPD presented a more nuanced association with GD. While individuals with comorbid GD and OCPD demonstrated lower gambling severity and slower progression to disorder onset, they nonetheless exhibited clinically significant gambling problems and a higher prevalence of substance use disorders [33]. This pattern may reflect the characteristic features of obsessive–compulsive personality disorder, such as rigidity, perfectionism, and heightened need for control, which could initially limit impulsive gambling behaviors and delay escalation to severe gambling disorder. However, once gambling behavior becomes established, these same traits may contribute to persistent and maladaptive gambling patterns, driven by compulsivity rather than impulsivity. In this context, gambling may function as a repetitive, rule-bound behavior that aligns with the cognitive style of individuals with OCPD, making cessation particularly difficult despite lower overall severity. The elevated prevalence of substance use disorders may further indicate the use of external regulators to manage emotional distress, frustration, or cognitive inflexibility, highlighting a distinct clinical profile in which gambling-related harm emerges through chronicity and comorbidity rather than rapid escalation.

Importantly, the review revealed substantial variability in prevalence estimates of PDs across studies, largely driven by differences in assessment methodology [35,36]. Interview-based studies employing validated diagnostic instruments tended to report higher absolute prevalence rates, reflecting systematic and comprehensive assessment of personality pathology, as reported in the Results section (Section 3.3; Table 3). In contrast, large register-based studies yielded lower recorded prevalence but demonstrated markedly elevated incidence ratios relative to the general population [36] (Table 2). This apparent discrepancy can be understood within a coherent methodological framework: registry-based diagnoses capture only documented cases, are influenced by healthcare access and diagnostic practices, and apply higher diagnostic thresholds, whereas interview-based assessments detect subthreshold and previously unrecognized pathology. Taken together, these complementary approaches converge in indicating a robust association between gambling disorder and personality pathology, rather than contradictory findings.

### 4.2. Limitations

Several limitations should be considered when interpreting the findings of this review. First, the small number of studies and databases included and the majority of cross-sectional designs, precluding causal inferences regarding the temporal relationship between PDs and GD. The search was conducted in only two databases using a limited set of search terms; consequently, studies indexed mainly in psychology databases or using specific personality disorder labels, may have been missed, and future searches incorporating additional PD-specific terms could retrieve further records. This review focuses on a single underlying clinical phenomenon: the presence of diagnosed PDs or personality disorder–level pathology in individuals with GD. This deliberate restriction to diagnosed or diagnostically anchored personality symptomatology also explains the relatively small number of eligible studies identified. However, it remains unclear whether personality pathology predisposes individuals to GD, emerges as a consequence of chronic gambling behavior, or reflects shared vulnerability factors.

Second, substantial heterogeneity was observed in diagnostic criteria and assessment instruments used to evaluate both GD and personality pathology. Studies relied on different versions of structured clinical interviews, and self-report questionnaires, which may yield divergent prevalence estimates and limit comparability across studies. However, it is important to note that all included articles employed validated assessment instruments for GD and PDs, each grounded in established diagnostic criteria. Thus, while methodological heterogeneity exists, the assessments remained conceptually and diagnostically consistent across studies, supporting the clinical relevance of the synthesized findings. An additional methodological limitation concerns the use of the SOGS as a proxy for DSM-based diagnostic criteria for GD in one included study [35]. This limitation should be considered when interpreting the overall conclusions of this study, particularly with regard to clinical implications.

Third, the majority of included studies were characterized by a marked predominance of male participants, which limits the generalizability of the findings to female populations and may obscure gender-specific patterns of comorbidity. As a result, it remains unclear whether the observed associations between GD and PDs operate similarly across genders or whether distinct clinical profiles may exist among women, underscoring the need for future research with more gender-balanced samples and explicit gender-sensitive analyses. Additionally, cultural and geographical differences across study populations may further influence personality expression and gambling behavior.

### 4.3. Implications for Clinical Practice and Future Research

The findings of this review have important clinical implications. The assessment of personality pathology in individuals with GD may enhance diagnostic accuracy, improve risk stratification, and facilitate the development of more personalized treatment approaches. From a therapeutic perspective, integrating interventions targeting emotional regulation, impulse control, and interpersonal functioning may be particularly beneficial for patients with comorbid personality pathology.

Future research should prioritize longitudinal designs to clarify causal pathways between PDs and GD. Greater methodological standardization in diagnostic assessment, along with the use of both categorical and dimensional personality models, would enhance comparability across studies. Additionally, further investigation into gender differences, cultural factors, and treatment responsiveness among specific personality profiles is warranted to advance precision psychiatry in GD.

## 5. Conclusions

This systematic review highlights the substantial overlap between GD and personality pathology, emphasizing the clinical relevance of maladaptive personality traits in individuals with GD. The evidence indicates elevated rates of antisocial and borderline personality disorders alongside heightened impulsivity and emotional dysregulation, which are associated with greater gambling severity and poorer functional outcomes. At the same time, the findings underscore the heterogeneity of personality profiles in GD, with certain traits, such as obsessive–compulsive and narcissistic personality characteristics, demonstrating more complex and context-dependent associations.

Future studies employing standardized diagnostic approaches and longitudinal designs are essential to clarify causal relationships and to advance the development of personalized, evidence-based interventions for GD.

## Figures and Tables

**Table 1:** Characteristics of the Included Studies.

Study Identification	Country	Study Design	Sample (*n*)	M/F Distribution	Age (Mean ± SD) Years	Diagnostic Criteria	Type of Gambling
Medeiros et al., 2018 [33]	USA	Cross-sectional study	50 (25 GD + obsessive–compulsive personality disorder (OCPD); 25 GD without any PD, matched by age and gender)	44% males (*n* = 22), 56% females	44.8 ± 11.8	DSM-5	Mix of gambling activities; card games less frequent in GD + OCPD; both groups reported use of electronic gaming machines, sports betting, lottery.
Fierro et al., 2024 [34]	Spain	Observational study	71 GD patients	91.5% males (*n* = 65); 8.5% females *(n* = 6)	Age groups: 20–30: 25.4%; 31–40: 23.9%; 41–50: 26.8%; >50: 23.9%	DSM-5	Land-based gambling 70.4%; Online gambling 25.4%; Unknown 4.2%
Maniaci et al., 2017 [35]	Italy	Longitudinal observational study (6-month follow-up)	194 (pathological gamblers (PG); 74 (controls)	PGs: 88.6% males (172 M/22F) Controls: 82.4% males (61 M/13F)	PGs: 42.21 ± 11.92 Controls: 41.54 ± 13.33	South Oaks Gambling Screen (SOGS) with high correlations with DSM-IV criteria	Multiple types: - sports betting (68%), - slot machines (58%), - lotteries (52%), - card games (22%), - bingo (16%) - bet on animals (8%) - dice games (6%) - casinos (6%)
Salonen et al., 2025 [36]	Finland	Nationwide register-based observational study (2011–2022)	3605 adults with clinically diagnosed GD	2574 males 1031 females	35.4 (range 18–90+)	ICD-10	Various gambling types (unspecified)
Quilty et al., 2021 [37]	Canada	Cross-sectional study	245 participants: 60 non-gamblers; 111 social gamblers; 35 non-treatment-seeking pathological gamblers; 39 treatment-seeking pathological gamblers.	130 males 115 females	41.86 ± 12.86	DSM-IV	Various gambling types (unspecified)
Rogier et al., 2018 [38]	Italy	Cross-sectional study	178 (74 GD and 105 controls)	GD group: 84.9% males; Controls: 76.2% males.	47.24 ± 11.64	DSM-5	Mixed: strategic (sports betting, animal betting, card games) and non-strategic (slot machines, scratch cards)
Moon et al., 2016 [39]	Canada	Cross-sectional study	150 pathological gamblers	75 M (50%), 75 F (50%)	36.3 ± 15.6	DSM-IV	Various gambling types (unspecified)
Black et al., 2021 [40]	USA	Cross-sectional study	285 pathological gamblers	54.7% males; 45.3% females	47.1 ± 17.7	DSM-IV	Game preference: - slots (39.1%) - action games (36.8%) - other games (24.1%)

**Table 2:** Main Findings in Personality Disorders Assessment Based on Diagnostic and Statistical Manual for Mental Disorders (DSM)/International Classification of Diseases, Tenth Revision (ICD-10) Diagnostic Criteria.

Study Identification	Personality Assessment Tool	Instrument Description	Other Measures	Main Findings/Key Outcomes Related to PD–GD Relationship
Medeiros et al., 2018 [33]	Structured Clinical Interview for DSM-IV Axis II Personality Disorders (SCID-II)	Structured diagnostic interview assessing all DSM-IV personality disorders.	Gambling Symptom Assessment Scale (G-SAS) Clinical Global Impression Scale (CGI) Mini-International Neuropsychiatric Interview (MINI) Minnesota Impulsive Disorders Interview Hamilton Anxiety Scale (HAM-A) and the Hamilton Depression Rating Scale (HAM-D).	- GD + obsessive–compulsive personality disorder (OCPD) group showed lower GD severity and slower progression from recreational gambling to GD. Nonetheless, it is important to notice that participants with co-occurring OCPD still demonstrated a considerably high severity of gambling problems. - The GD + OCPD group, when compared to the group without OCPD, reported more triggers to gambling (money availability, stress, loneliness, advertisements). - However, the group GD + OCPD demonstrated higher prevalence of substance-use disorders.
Salonen et al., 2025 [36]	ICD-10 clinical diagnoses (F60–F69) recorded in national healthcare registers.	PD diagnoses based on ICD-10 criteria assigned by healthcare professionals in primary and specialized care.	All psychiatric comorbidities from ICD-10 codes (F00–F99)	- Of individuals with GD, 7.9% were diagnosed with adult personality disorder. Furthermore, 22.7% had a behavioral syndrome associated with psychological disturbances and physical factors, 13.6% had schizophrenia spectrum disorder, and 12.3% had behavioral and emotional disorder with an early onset. Additionally, one in ten (10.5%) had attention deficit hyperactivity disorder (ADHD) spectrum diagnoses. - The highest standardized incidences in different diagnostic groups were for personality disorders (SIR = 616.6; 95% CI = 570.0–665.1). - Women: PD incidence extremely elevated (SIR = 839.0). - Men: PD also strongly elevated (SIR = 678.6).
Quilty et al., 2021 [37]	Structured Clinical Interview for DSM-IV Personality Disorders, Personality Questionnaire (SCID-II/PQ) Multi-source Assessment of Personality Pathology (MAPP) Diagnostic Interview for DSM-IV Personality Disorders (DIPD-IV)	SCID-II/PQ: self-report DSM-IV PD questionnaire. MAPP: informant-rated DSM-IV PD assessment. DIPD-IV: “gold standard” clinical interview for DSM-IV PDs. All scales varied in item format (SCID-II/PQ items are scored 0 or 1, DIPD items are scored 0, 1, or 2, and MAPP items are scored 1 to 4).	Structured Clinical Interview for DSM-IV, Axis I Disorders, Patient Form (SCID-I/P) Canadian Problem Gambling Questionnaire (CPGI) South Oaks Gambling Screen (SOGS) Revised NEO Personality Inventory (NEO PI-R) Structured Interview for the Five-Factor Model (SIFFM)	- PG group showed elevated symptoms on multiple PDs across measures: Borderline, Paranoid, Schizotypal, Avoidant, Dependent. - PGs also showed higher Antisocial, Narcissistic, and Obsessive–Compulsive PD symptoms.
Moon et al., 2016 [39]	Structured Clinical Interview (SCID-P) for DSM-IV Antisocial Personality Disorder	Semi-structured clinical interview assessing DSM-IV Antisocial Personality Disorder symptoms	Coping subscale of the Gambling Motives Questionnaire (GMQ) Childhood Trauma Questionnaire (CTQ) UPPS Impulsive Behavior Scale Harm Avoidance subscale of the Multidimensional Personality Questionnaire (MPQ) Delayed Discounting of Monetary Rewards Task Conners’ Adult ADHD Rating Scale (CAARS) National Opinion Research Center DSM-IV Screen for Gambling Problems (NODS) Canadian Problem Gambling Index (CPGI)	- Antisocial personality disorder symptoms were highest in the Antisocial–Impulsivist (AI) gambling subtype. - AI gamblers also showed greater impulsivity, risk-taking, and ADHD symptoms.
Black et al., 2021 [40]	Structured Interview for DSM-IV Personality (SIDP-IV)	Semi-structured clinical interviews assessing DSM-IV personality pathology, with a specific focus on antisocial personality disorder (ASPD) symptoms.	South Oaks Gambling Screen (SOGS) National Opinion Research Center DSM-IV Screen for Gambling Problems (NODS) Minnesota Impulsive Disorders Interview (MIDI) Structured Clinical Interview for DSM-IV (SCID-IV) Mini International Neuropsychiatric Interview-Attention Deficit Hyperactivity Disorder Module (MINI-ADHD) Barratt Impulsiveness Scale (BIS) Medical Outcome Study Short Form-36 (MOS-SF36) Gambling Symptom Assessment Scale (GSAS) Revised Childhood Experiences Questionnaire	- ASPD symptoms were most prevalent in the Antisocial Drinker (AD) and Antisocial-Impulsivist (AI) classes. - The AI subtype showed the highest levels of impulsivity, ADHD, and severe gambling. - Findings support the Pathways Model, demonstrating heterogeneity of personality dysfunction among individuals with GD.

**Table 3:** Main Findings in Diagnostically Anchored PD Symptomatology Based on Diagnostic and Statistical Manual for Mental Disorders (DSM)-Aligned Validated Instruments.

Study Identification	Personality Assessment Tool	Instrument Description	Other Measures	Main Findings/Key Outcomes Related to PD–GD Relationship
Fierro et al., 2024 [34]	Exploratory Personality Questionnaire-III (CEPER-III)	Instrument built and validated in Spain, based on the diagnostic symptoms of the DSM-IV-TR. 170-item measure assessing 14 personality domains: paranoid, schizoid, schizotypal, histrionic, narcissistic, antisocial, borderline, avoidant, dependent, obsessive-compulsive, passive-aggressive, sadistic, self-destructive, depressive.	International Physical Activity Questionnaire—short version, 9 items (IPAQ)	- GD patients showed lower scores than the general population on paranoid, histrionic, narcissistic, passive-aggressive, and sadistic traits. - High antisocial and high borderline scores predicted low physical activity; high obsessive-compulsive and high self-destructive scores predicted moderate/high activity.
Maniaci et al., 2017 [35]	Millon Clinical Multiaxial Inventory–III (MCMI-III)	175-item true/false self-report instrument assessing Axis I clinical syndromes and Axis II personality disorders (according to classication of DSM IV) based on Millon’s evolutionary theory.	South Oaks Gambling Screen (SOGS)	- High PD prevalence among GD patients: 59.8% had at least one PD; 25.6% had two or more PDs. - PGs scored significantly higher than controls on several PDs (Narcissistic, Antisocial, Negativistic, Self-Defeating). - Specific PDs predicted treatment dropout: Antisocial PD and Negativistic (Passive-Aggressive) PD significantly increased risk of early treatment dropout. - Other predictors: PTSD and Drug Dependence also predicted dropout. - Anxiety acted as a protective factor.
Rogier et al., 2018 [38]	Pathological Narcissism Inventory (PNI)	52-item self-report validated multidimensional measure (APA, 2013) developed to capture both grandiose and vulnerable expressions of narcissism precisely in addition to DSM-IV criteria for Narcissistic Personality Disorder	South Oaks Gambling Screen (SOGS) Difficulties in Emotion Regulation Scale (DERS)	- Individuals with GD showed significantly higher levels of both grandiose and vulnerable pathological narcissism compared to controls. - Grandiose narcissism predicted GD severity, and emotion dysregulation fully mediated the relationship between grandiose narcissism and GD severity. - Vulnerable narcissism was strongly associated with emotion dysregulation. - Strategic gamblers exhibited higher levels of devaluing (vulnerable narcissism) than non-strategic gamblers.

## Data Availability

The raw data supporting the conclusions of this article will be made available by the authors on request.

## Supplementary Materials

The following supporting information can be downloaded at: https://www.mdpi.com/article/10.3390/clinpract16010015/s1, File S1: PRISMA 2020 checklist.

## Abbreviations

The following abbreviations are used in this manuscript:

AD	Antisocial Drinker
ADHD	Attention Deficit Hyperactivity Disorder
AI	Antisocial–Impulsivist
ASPD	Antisocial Personality Disorder
BIS	Barratt Impulsiveness Scale
CAARS	Conners’ Adult ADHD Rating Scale
CEPER-III	Exploratory Personality Questionnaire-III
CGI	Clinical Global Impression Scale
CPGI	Canadian Problem Gambling Questionnaire
CTQ	Childhood Trauma Questionnaire
DERS	Difficulties in Emotion Regulation Scale
DIPD-IV	Diagnostic Interview for DSM-IV Personality Disorders
DSM	Diagnostic and Statistical Manual of Mental Disorders
GD	Gambling Disorder
GMQ	Gambling Motives Questionnaire
GSAS	Gambling Symptom Assessment Scale
HAM-A	Hamilton Anxiety Scale
HAM-D	Hamilton Depression Rating Scale
ICD-10	International Classification of Diseases, Tenth Revision
IPAQ	International Physical Activity Questionnaire
JBI	Joanna Briggs Institute
MAPP	Multi-source Assessment of Personality Pathology
MCMI-III	Millon Clinical Multiaxial Inventory–III
MIDI	Minnesota Impulsive Disorders Interview
MINI	Mini-International Neuropsychiatric Interview
MINI-ADHD	Mini International Neuropsychiatric Interview—Attention Deficit Hyperactivity Disorder Module
MOS-SF36	Medical Outcome Study Short Form-36
MPQ	Multidimensional Personality Questionnaire
NEO PI-R	Revised NEO Personality Inventory
NODS	National Opinion Research Center DSM-IV Screen for Gambling Problems
NOS	Newcastle–Ottawa Scale
OCPD	Obsessive–Compulsive Personality Disorder
PDs	Personality Disorders
PG	Pathological Gambling
PNI	Pathological Narcissism Inventory
PRISMA	Preferred Reporting Items for Systematic Reviews and Meta-Analyses
SCID-I/P	Structured Clinical Interview for DSM-IV, Axis I Disorders, Patient Form
SCID-II	Structured Clinical Interview for DSM-IV Axis II Personality Disorders
SCID-II/PQ	Structured Clinical Interview for DSM-IV Personality Disorders, Personality Questionnaire
SD	Standard Deviation
SIDP-IV	Structured Interview for DSM-IV Personality
SIFFM	Structured Interview for the Five-Factor Model
SOGS	South Oaks Gambling Screen
